# Thrombolysis of Postoperative Acute Pulmonary Embolism with a Thrombus in Transit

**DOI:** 10.1155/2020/7561986

**Published:** 2020-05-19

**Authors:** Cynthia Mardinger, Paul J. E. Boiteau, John B. Kortbeek

**Affiliations:** ^1^Cumming School of Medicine, University of Calgary, Foothills Medical Centre, 3330 Hospital Dr NW, Calgary, AB, Canada T2N 4N1; ^2^Department of Critical Care Medicine, Cumming School of Medicine, University of Calgary, 3330 Hospital Dr NW, Calgary, AB, Canada T2N 4N1; ^3^Departments of Surgery and Anaesthesia, Cumming School of Medicine, University of Calgary, 3330 Hospital Dr NW, Calgary, AB, Canada T2N 4N1

## Abstract

Right heart thrombus in transit clot (RHTT) associated with a pulmonary thromboembolism (PTE) is a rare but potentially fatal diagnosis. Early diagnosis and immediate intervention are crucial. This report describes the case of a healthy, physically active 32-year-old female who presented 19 days postoperatively, following an anterior cruciate ligament reconstruction and partial lateral meniscectomy with a saddle PE, RHTT, and right ventricular (RV) strain. The patient received half of the standard dose of intravenous tissue plasminogen activator (TPA) in combination with anticoagulation and survived. Case reports of RHTT will inform future studies designed to evaluate whether and when thrombolysis should be administered.

## 1. Introduction

Right heart thrombus in transit clot (RHTT) is a rare complication of venous thromboembolism that occurs in up to 4% of patients with pulmonary embolism (PTE) with a very high mortality [1]. It has been reported to be uniformly fatal in untreated patients and in 5.7–27% who receive treatment, placing patients at a significantly higher mortality risk than those with an acute PTE alone [2–6]. Therefore, patients who develop RHTT may be considered for reperfusion therapy.

In this report, we describe the presentation, management, and outcome of a patient who presented with a saddle PTE, RHTT, and right ventricular (RV) strain who received half of the standard dose of intravenous tissue plasminogen activator (tPA) in combination with anticoagulation.

## 2. Case Presentation

A fit, physically active 32-year-old female with no underlying risk factors underwent a left knee anterior cruciate ligament (ACL) reconstruction and partial lateral meniscectomy. She was not taking oral contraceptives. She presented 19 days postoperatively with acute dyspnea, chest pain, tachycardia, and complaints of feeling light-headed of 5 days duration. In the emergency department, she had sinus tachycardia on electrocardiography, a heart rate of 126 bpm, blood pressure of 107/68 mmHg, respiratory rate of 18/min, and an oxygen saturation of 95% on room air. Her examination was noted to be otherwise unremarkable. There was no evidence of surgical site infection and no limb swelling noted. A complete blood count and electrolyte and metabolic panel were normal. Her troponin was 80 ng/L (normal 0–14 ng/L). A chest radiograph and D-dimer were not performed. Her pretest probability of pulmonary embolism was moderate based on Wells' criteria [7].

**Figure 1:**
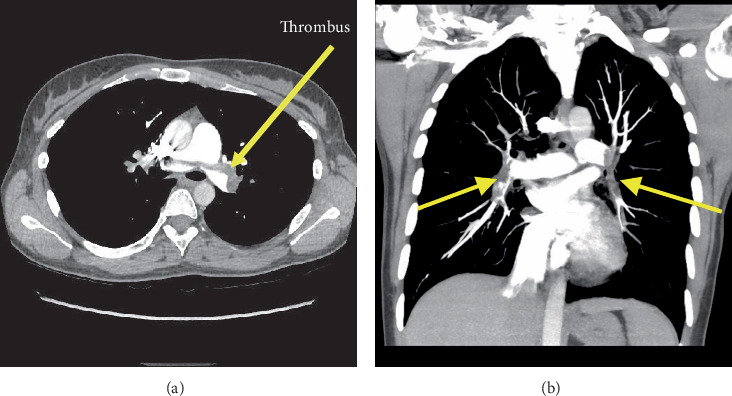
Computed tomography pulmonary angiogram (CTPA). (a) Axial image. Low-density (grey) filling defect noted across the main pulmonary arterial trunk at its bifurcation. (b) Coronal image. Bilateral pulmonary emboli extending into all lobar and segmental branches. Arrows identify thrombus.

Computed tomography pulmonary angiogram (CTPA) reported the patient as having PTE. Findings included saddle PE, extensive clot extending into lobar and segmental branches of all lobes (Figure 1) as well as evidence of right heart strain with marked right atrial (RA) and RV dilation as well as flattening and deviation of the interventricular septum. The RV/LV ratio was 1.3. Unfractionated low-molecular-weight heparin was immediately initiated. Her simplified PE Severity Index (sPESI) was 1 (high risk, 8.9% mortality) [8].

**Figure 2:**
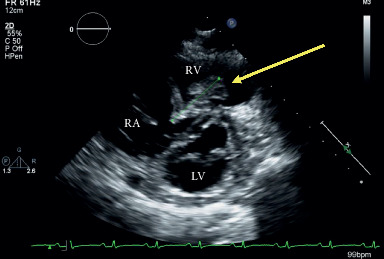
Parasternal short-axis view at the mitral valve level, demonstrating a dilated right ventricle, a D-shaped interventricular septum, and a large multilobulated mass in the right ventricle (arrow). RA: right atrium; RV: right ventricle; LV: left ventricle.

The patient was admitted to the Internal Medicine service. On day 1, her clinical status was unchanged; laboratory indices were similar with a troponin level of 35 ng/L and NTpro-BNP of 3859 ng/L (normal 0–300). ICU was consulted on day 1 of admission, and a transthoracic echocardiogram (TTE) was immediately requested. This demonstrated a 3.3 cm maximum diameter multilobulated, mobile right atrial thrombus, which extended from the RV inlet through the pulmonic valve to the pulmonary artery (i.e., an RHTT). Intraventricular septal flattening and severe RV dilation with moderate systolic dysfunction and tricuspid regurgitation were also observed. McConnell's sign was present with apical hypercontractility and basal and mid-ventricular segmental hypokinesis. The right ventricular systolic pressure (RVSP) was 48.1 mmHg (Figure 2).

**Figure 3:**
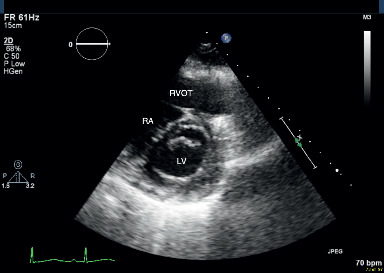
Parasternal short-axis view at the mitral valve level, performed on the day following thrombolysis. Note the absence of both the thrombus and the D-shaped interventricular septum. RA: right atrium; RVOT: right ventricular outflow tract; LV: left ventricle.

She was transferred to the intensive care unit (ICU) with a diagnosis of clinical submassive pulmonary embolism with clot in transit. Arterial blood gas was pH 7.52, pCO_2_ 29 mmHg, PaO_2_ 74 mmHg, O_2_ saturation 96%, and lactate 0.8 mmol/L. The case, as well as treatment options, was reviewed with a member of the pulmonary embolism response team (PERT). A decision was made to proceed with low-dose thrombolysis, despite the recent surgery, employing the regimen described in the MOPETT trial [9]. The potential benefits and risks were discussed with the patient who consented. She received a 10 mg recombinant tPA bolus followed by an additional 40 mg over two hours intravenously (IV). IV heparin was continuously infused at 10 U/kg/hr during the thrombolysis as well and for three hours afterwards at which time the heparin nomogram infusion of 18 U/kg/hr with serial adjustments based on PTT was re-introduced. Serial echocardiograms at 2 hours after thrombolysis and the next day (Figure 3) demonstrated improvement in her RV function. The RVSP was 27 mmHg at 2 hours and 26 mmHg the following day. The in-transit clot was no longer visible on the posttreatment echocardiograms. She was eventually discharged home on day 5 on tinzaparin for two weeks and then transitioned to rivaroxaban. She was continued on anticoagulation for 6 months.

Seven months after discharge, she was doing very well. Her breathing had returned back to baseline, and she was rehabilitating well from her reconstruction. She had no bleeding complications from the rivaroxaban therapy. Her DASH score [10] was 1 (3.9% annual risk of DVT recurrence), and her HERDOO2 score [11] was 0 (3% risk of major recurrent venous thromboembolism per 100 patient years). The anticoagulation was discontinued. She had returned to the gym, though had not yet returned to more vigorous-intensity physical activities, such as basketball. A repeat echocardiogram at this time demonstrated normal left and right ventricular systolic function with no hemodynamically significant valvular disease. Serial D-dimers, every three months, for the following year were scheduled. Testing for inherited thrombophilia was negative.

## 3. Discussion

Though management of RHTT has not been well established in the literature, current treatment modalities include anticoagulation therapy, thrombolytic therapy, catheter-directed thrombolysis, and surgical embolectomy [3]. RHTT is uncommon and management decisions are often based on risk stratification and center expertise, as there are currently no randomized control trials comparing treatment options. Nonetheless, studies suggest anticoagulation therapy is insufficient on its own, particularly when high-risk features including large size and extent of the in-transit clot (free floating), as well as submassive PE, are present [5, 12]. Others have suggested no mortality benefit with thrombolysis [4]. Assessment by multidisciplinary teams including intensivists and pulmonary embolism response teams may assist in recommending treatment for individual patients.

Systemic thrombolysis has been reported to have major bleeding risks including potentially fatal intracranial haemorrhage rates of 0.7–6.4% [9]. Recent surgery, as in this case, is a major risk factor for bleeding when administering thrombolysis, with rates of up to 25% for surgical site bleeding [13]. Surgical embolectomy and older studies using catheter-directed therapies have demonstrated a higher risk of mortality compared to medical thrombolytic management [3]. The anatomic area and magnitude of surgery as well as the postsurgical interval are factors that should be considered when estimating the risks of thrombolysis.

The Moderate PE Treated with Thrombolysis (MOPETT) trial is a randomized controlled trial of thrombolysis versus standard anticoagulation. The study demonstrated that patients who received half-dose systemic thrombolysis (i.e., 50 mg of tPA instead of 100 mg) as an adjunct to anticoagulation in the setting of “submassive” PE (RV strain on TTE) had a reduction in the combined endpoints of death and recurrent PE, a shorter hospital stay, and a significant immediate reduction in pulmonary arterial pressure. This was maintained beyond two years in comparison to patients treated with anticoagulation therapy alone [9]. No bleeding occurred in either group. Studies have suggested that the bleeding risk with thrombolytics may historically have been overestimated [14–17].

Current guidelines by the American Heart Association define massive, submassive, and low-risk PE based on likelihood of mortality [18]. A massive PE is one that causes sustained hypotension (<90 mmHg systolic for >15 minutes) or requires vasopressor support. The European Society of Cardiology and the European Respiratory Society recently published guidelines refer to this group of patients as high risk [19]. These patients can go on to develop cardiac arrest or persistent bradycardia (heart rate <40 bpm with signs and symptoms of shock) without any apparent causes. They also typically exhibit 30-day fatality rates of 20–40% or greater and require both reperfusion therapy and immediate treatment of acute right heart failure [20].

A submassive PE is one that causes right heart strain, dilation, dysfunction, or ischemia [18]. RV function (strain or dysfunction) can only be assessed by echocardiography, whereas dilation (RV/LV ratio of >0.9) can also be assessed through CTPA imaging. Elevations in brain natriuretic peptide or troponin can also qualify a PE as submassive. These patients can develop new complete or partial right bundle branch block, anteroseptal ST changes, or anteroseptal T-wave inversions seen on electrocardiography. To better risk stratify this group of patients, the European Society of Cardiology and the European Respiratory Society Guidelines stress the importance of RV dysfunction on TTE or CTPA in combination with elevated cardiac biomarkers despite hemodynamic stability (systolic BP >90 mmHg and no need for vasopressors) to define an intermediate high risk from an intermediate low-risk group of patients. The intermediate high-risk group of patients should be admitted to a unit with close monitoring (i.e., ICU) to permit the early detection of hemodynamic decompensation or collapse and the need for rescue reperfusion therapy [19]. Finally, low-risk PE is defined as having no associated hypotension or right heart dysfunction or dilation [18].

Although on initial presentation our patient was deemed to be stable, her shock index (HR/systolic BP) was elevated to 1.18 (normal ≤0.7) and she exhibited both imaging and biochemical evidence of RV dysfunction. This presentation would have her classified as an intermediate high-risk patient in need of close observation. Her shock index on presentation was an indicator of circulatory dysfunction and should be a trigger to better assess myocardial performance with a TTE [21].

In general, right heart thrombus in the setting of PE is uncommon. There are previous existing case reports of using half-dose thrombolysis to successfully treat an in-transit clot with complete resolution [22, 23]. A recent study comparing the efficacy of different treatment modalities for right heart thrombi in transit reported thrombolysis was more effective than anticoagulation alone for improving survival without any additional complication risks [24]. This case further illustrates the potential utility of half-dose thrombolysis in combination with anticoagulation in the successful resolution of a submassive PE complicated by a RHTT. This case also describes the rare usage of thrombolysis in the postoperative period, within 30 days of surgery.

Given that this patient had orthopaedic surgery and had spinal anaesthesia, this may suggest this was a provoked clot. However, some guidelines advocate that only major orthopaedic surgery or general anaesthesia be considered provoked [25]. Although her PE occurred following orthopaedic surgery, ACL reconstruction is generally felt to be low risk of embolic complications. Current guidelines do not recommend VTE prophylaxis for these procedures [26]. Although the patient was not at her baseline level of activity, she had been discharged home on the day of surgery, was mobilizing, and had already started rehabilitation prior to developing a clot. She had no significant immobility and no other risk factors, aside from her surgery.

Current recommendations by the American College of Chest Physicians suggest patients with a PE should receive treatment with anticoagulation for three months, regardless of whether or not it was provoked by surgery [26]. Three months is preferred over a treatment of a shorter period, a longer time-limited period (e.g., 6, 12, or 24 months), or extended therapy (no scheduled stop date). However, for patients with a first VTE that is unprovoked and who have a low or moderate bleeding risk, extended anticoagulation therapy is preferable over three months of therapy. For those with high bleeding risk, three months of anticoagulation therapy is preferred over extended therapy.

Given our patient had a weakly provoked clot that was submassive in the context of no previous history of venous thromboembolism, there are no clear guidelines on the overall duration of anticoagulation therapy. In general, guidelines have not changed the recommendations based on overall size of clots, though some experts have advocated long-term therapy for massive PE. However, there is currently no significant evidence to support this practice [20]. Using risk scores for unprovoked clots, the patient had low DASH and HERDOO2 scores, which also suggested anticoagulation could be discontinued [10, 11].

Bedside ultrasound of the femoral veins was performed by the critical care team (negative for thrombus), but the patient did not undergo formal duplex ultrasonography by the Diagnostic Imaging Department to exclude deep venous thrombosis (DVT). This should be considered in similar cases as patients with isolated PTE without DVT may represent a high-risk subgroup [27].

This patient will have a high lifetime risk of recurrent clotting events. However, there is currently no effective way of predicting if or when she would develop unprovoked recurrence. Following discussion of the equivocal current evidence of continuing anticoagulation, the patients' preference was to discontinue therapy. The role of serial D-dimers was discussed, and the patient was amenable to undergoing monthly D-dimers for a subsequent three months. If she had persistent positive D-dimers, consideration would be given to resuming anticoagulation. An increased risk of recurrent venous thromboemboli after discontinuing anticoagulation with vitamin K antagonists persists for up to 4 months. This may reflect a hypercoagulable state after discontinuing anticoagulation and is associated with elevated D-dimer levels [28]. In patients with a higher risk of recurrent venous thromboemboli (e.g., unprovoked clot), extended therapy with reduced-dose direct oral anticoagulants (rDOAC) may be a consideration. Recent clinical trials have suggested that rDOAC are equivalent to full-dose therapy in reducing recurrent venous thromboemboli, yet are associated with similar risks of bleeding to placebo [29].

Current guidelines also do not advocate thrombophilia testing since it does not significantly change duration of therapy or risk of recurrence except in cases of antiphospholipid antibody (APLA) syndrome [30]. However, given the size of her clot, the patient chose to undergo further testing.

In conclusion, RHTT associated with a PE is a rare but potentially fatal diagnosis. Early diagnosis and immediate intervention are crucial. Our case demonstrates successful treatment of a thrombus in transit with half-dose thrombolysis in combination with intravenous unfractionated heparin anticoagulation. Additional case reports will inform future studies. These are necessary to evaluate whether and when this treatment should be implemented with good efficacy and lower risk than other treatment modalities.
